# Controlling Molecular Dye Encapsulation in the Hydrophobic Core of Core–Shell Nanoparticles for In Vivo Imaging

**DOI:** 10.1007/s44174-023-00073-0

**Published:** 2023-04-07

**Authors:** Masakazu Umezawa, Yuichi Ueya, Kotoe Ichihashi, Doan Thi Kim Dung, Kohei Soga

**Affiliations:** 1 Department of Materials Science and Technology, Faculty of Advanced Engineering, Tokyo University of Science, 6-3-1 Niijuku, Katsushika, Tokyo 125-8585 Japan; 2 Tsukuba Research Laboratories, JSR Corporation, 25 Miyukigaoka, Tsukuba, Ibaraki 305-0841 Japan

**Keywords:** Polymeric nanoparticles, Polarity, Stability, Near-infrared, Second biological window

## Abstract

Polymeric nanoparticles with a hydrophobic core are valuable biomedical materials with potential applications in in vivo imaging and drug delivery. These materials are effective at protecting vulnerable molecules, enabling them to serve their functions in hydrophilic physiological environments; however, strategies that allow the chemical composition and molecular weight of polymers to be tuned, forming nanoparticles to control the functional molecules, are lacking. In this article, we review strategies for designing core–shell nanoparticles that enable the effective and stable encapsulation of functional molecules for biomedical applications. IR-1061, which changes its optical properties in response to the microenvironment are useful for in vitro screening of the in vivo stability of polymeric nanoparticles. An in vitro screening test can be performed by dispersing IR-1061-encapsulated polymer nanoparticles in water, saline, buffer solution, aqueous protein solution, etc., and measuring the absorption spectral changes. Through the screening, the effects of the polarity, molecular weight, and the chiral structure of polymers consisting of polymer nanoparticles on their stability have been revealed. Based on the findings presented here, more methodologies for the effective application of various biomolecules and macromolecules with complex high-dimensional structures are expected to be developed.

## Molecule-Encapsulated Nanoparticles for Biomedical Applications

Nanoparticles are important in nanotechnology owing to their specific physicochemical properties and biological effects compared to those of larger particles [1]. In the biomedical field, nanoparticles are attracting attention as carriers to protect molecules from the external environment and transport and release them into target tissues and organs [2]. As drug-delivery systems, in vivo drug distribution is more controllable when nanoparticles of polymeric micelles and liposomes encapsulating drug molecules are used [3], thereby reducing side effects and enhancing drug efficacy [4]. For example, NK105, a polymeric micelle containing paclitaxel in its hydrophobic core, shows an approximately 25-fold higher tumor accumulation compared to free paclitaxel [5] and has been tested in clinical trials to evaluate its safety and efficacy in metastatic or recurrent breast cancer patients [6]. In 2020, nanoparticle-type vaccines were developed to gain effective immune induction to mitigate the coronavirus disease 2019 (COVID-19) pandemic by encapsulating mRNA with low in vivo stability in lipid nanoparticles [7, 8]. In the field of bioimaging, nanoparticles with contrast capability in magnetic resonance and fluorescence have been studied to obtain qualitative and quantitative spatiotemporal information of intracellular and in vivo structures [9]. Nanoparticles encapsulating both drugs and contrast agents are promising materials for the simultaneous treatment and diagnosis of diseases, which is called theranostics [10]. Thus, molecule-encapsulated nanoparticles are essential for the development of next-generation medical and healthcare industries. Research toward their design and development is currently underway.

Among the nanoparticles with a hydrophilic surface, which allows their stable existence in vivo, micellar nanoparticles can encapsulate poorly soluble molecules in their hydrophobic core [11]. Therefore, core−shell nanoparticles have been investigated for better applications in drug delivery [12, 13]. The hydrophobic core can be used to stably deliver highly degradable molecules in vivo, such as mRNA, to physiological environment. Contrast-enhancing molecules such as fluorescence dyes can also be encapsulated in a biocompatible hydrophilic shell to visualize in vivo structures and phenomena. Knowledge of the control mechanism of encapsulation is essential for harnessing the unique functions of the encapsulated molecules. Recently, methods for designing core–shell nanoparticles have been investigated to achieve a high level of fluorescence of fluorescent dye molecules encapsulated in the hydrophobic core.

## Near-Infrared Fluorescence Materials

Bioimaging is a technology used in biomedical and clinical fields to visualize the anatomical structures, biomarkers, and physiological features of living organisms in real time [14]. Compared with other modalities of anatomical and molecular imaging, such as X-ray, magnetic resonance imaging (MRI), positron emission tomography, single-photon emission computed tomography [15], and ultrasonography [16], fluorescence imaging has the advantages of no ionizing radiation exposure as well as high sensitivity and temporal resolution. Bio-applications of fluorescence imaging are hampered mainly by the limitations of observation depth owing to the effects of light scattering and absorption by biological tissues [17, 18]. Although it is difficult to obtain pathophysiological information at the whole-body level using visible light (wavelength range of 380−700 nm) owing to its limited biological transparency of less than 2−3 mm, near-infrared (NIR) light (wavelength range of 700−2500 nm) is being used for deep in vivo imaging owing to its high transparency in biological tissues [18]. For example, fluorescent dyes, such as indocyanine green (ICG) and methylene blue, which work in the NIR-I wavelength range (700−900 nm), have been clinically applied as contrast agents for blood and lymphatic vessels [19, 20]; however, their observation depth is still less than 10 mm. On the other hand, in the >1000 nm (over-thousand-nanometer: OTN) NIR region, even though the optical absorption owing to water molecular vibration is larger than that in the conventional NIR-I region, the reduction in scattering by biological tissues [21, 22] improves the observation depth to several centimeters [18].

Research has been conducted on the development of both imaging devices [23–25] and fluorescence probes [26] to realize the biomedical applications of OTN-NIR fluorescence imaging. Charge-coupled devices (CCD) and complementary metal-oxide-semiconductor (CMOS) cameras have been widely used in conventional fluorescence imaging devices. However, silicon-based detectors have very low quantum yields in the OTN region and are unsuitable for OTN-NIR fluorescence imaging. With the progress of optical communication technology, short-wavelength infrared (SWIR) cameras, which are sensitive to OTN-NIR and based on semiconductor alloys with narrow bandgaps (e.g., InGaAs, HgCdTe) [22] became commercially available in ca. 2005. Since then, OTN-NIR fluorescence imaging devices equipped with SWIR cameras have been commercialized.

**Fig. 1:**
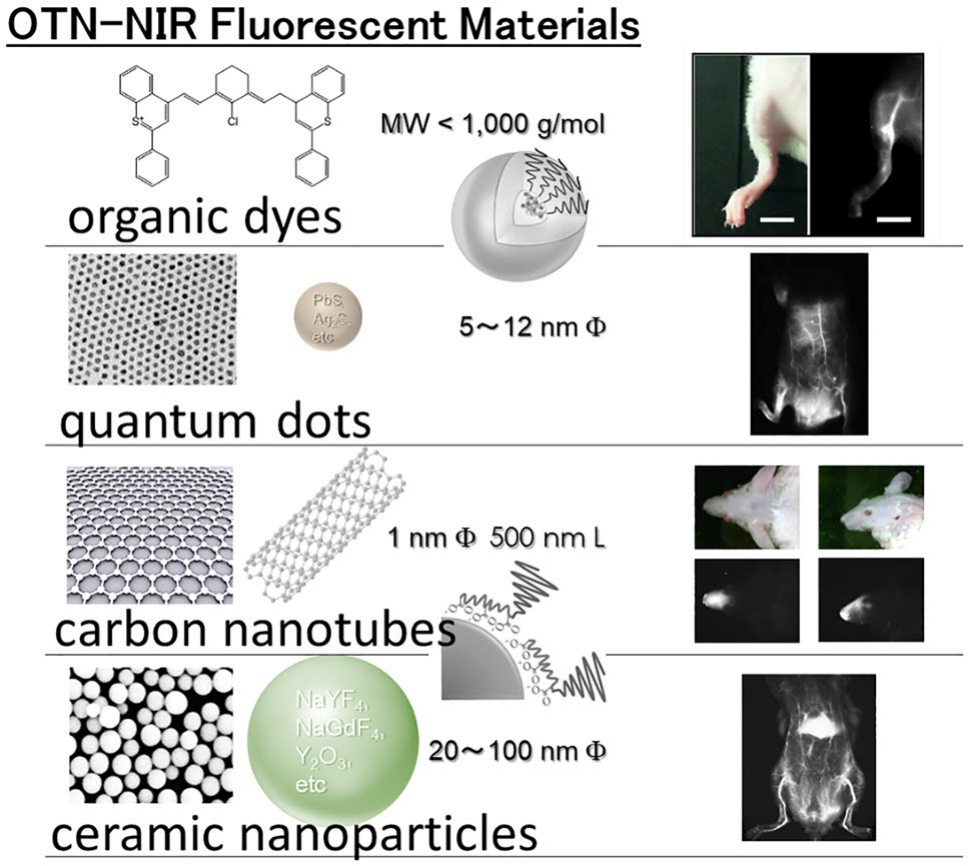
Typical OTN-NIR fluorescent probes

Phosphors for OTN-NIR fluorescence imaging include single-walled carbon nanotubes (SWCNTs) [23, 27–30], quantum dots [31, 32], rare-earth-doped ceramic nanoparticles (RED-CNPs) [33–39], and organic dyes [40–43] (Fig. 1). The optical properties of SWCNTs in NIR were reported in 2002 [44] and their first application in OTN-NIR fluorescence imaging was reported in 2009 [27]. SWCNTs are composed of carbon–carbon bonds that are chemically and thermally stable but have a special shape with a high aspect ratio, a diameter of less than several nanometers, and a length of several hundred nanometers. This unique characteristic has sparked interest in the in vivo pharmacokinetics of SWCNTs [45]. Quantum dots have high quantum yields and high chemical stabilities; however, their toxicity is a matter of concern owing to their inclusion of heavy metal elements such as cadmium and lead. RED-CNPs are inorganic ceramic nanoparticles doped with trivalent rare-earth ions with characteristic electron levels [24, 26]. These types of nanoparticles are promising as OTN-NIR fluorescence imaging contrast agents but are poorly degradable or excretable from tissues and organs. Hybrid nanostructures have been reported for ultrasmall RED-CNPs coupled with organic polymers to allow excretion after biodegradation [46]. However, the clinical application of RED-CNPs is still difficult, as they are solid nanoparticles. On the other hand, there is less concern about the safety of NIR fluorescent molecular dyes, as ICG and methylene blue have already been clinically applied. Moreover, the optical properties and molecular size can be easily controlled by tailoring the molecular structure, enabling the development of various applications.

## Near-Infrared Fluorescent Dyes

**Fig. 2:**
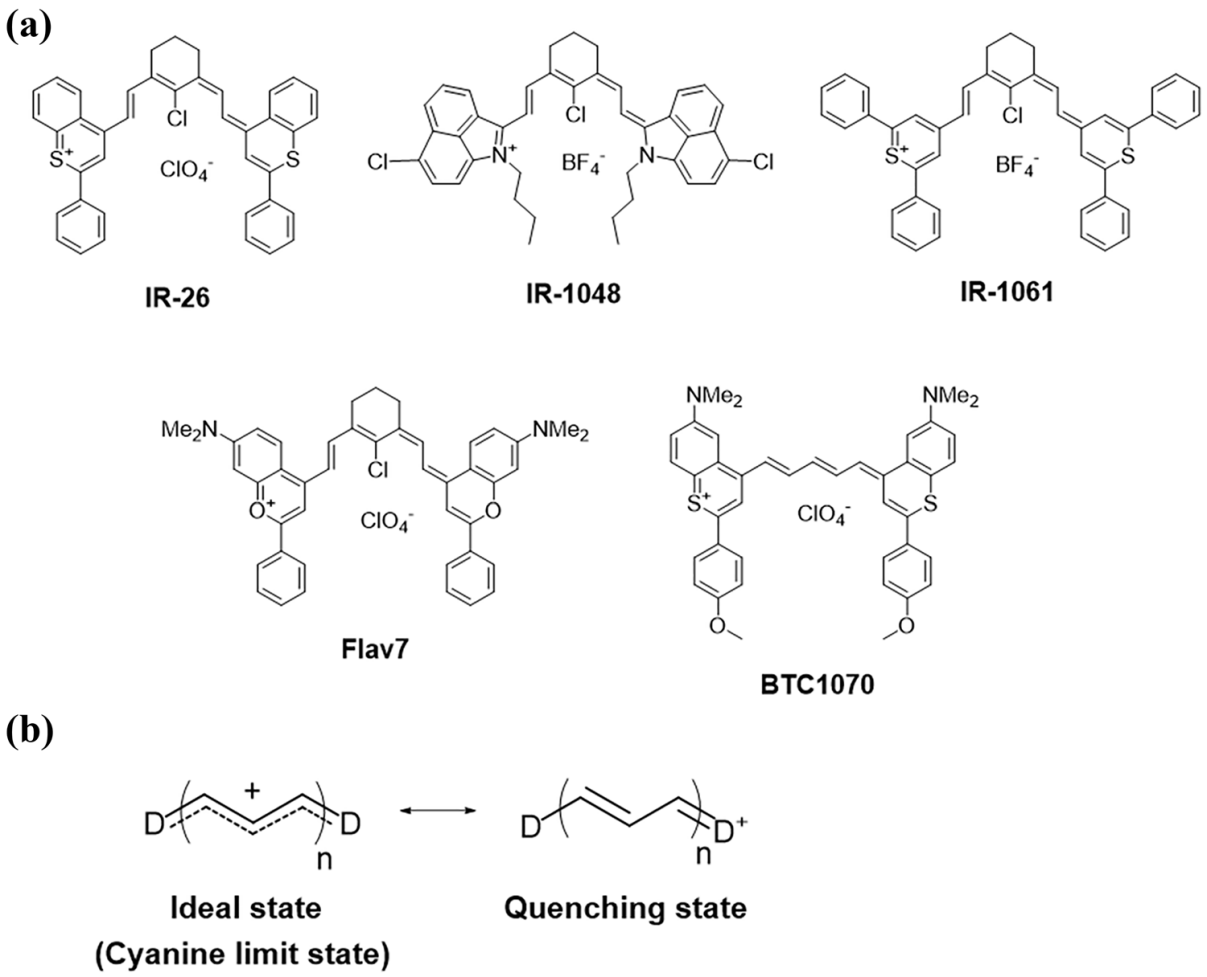
Structures and charge states of polymethine cyanine dyes. **a** Representative OTN-NIR dyes with polymethine backbone. **b** Two charge states of polymethine cyanine dye. D represents an electron-donating group

OTN-NIR fluorophores are classified into donor-acceptor-donor (DAD) dyes and polymethine dyes [47–49]. DAD dyes generally require high injection doses and fluence rates owing to their weak and short-wavelength absorption (*ε* ≈ 10^3^–10^4^ M^−1^ cm^−1^, *λ*_abs_ < 900 nm) [50]. On the other hand, polymethine dyes with polymethine chains and two heterocyclic terminal structures (Fig. 2a) have the advantage of a large absorption coefficient (*ε* > 10^5^ M^−1^ cm^−1^), and the absorption peak can be tuned to 1200 nm by molecular design [51]. Novel organic dyes that operate in the OTN-NIR wavelength range have also been developed. The main strategies for obtaining long-wavelength (i.e., OTN) NIR fluorescence at high quantum yields are the extension of the polymethine chain and an increase in the electron density of the donor in DAD and polymethine (D-π-A) dyes [50]. As described in published reports [52], NIR polymethine dyes can be chemically obtained by combining two heterocyclic molecules with increased reactivity by introducing a formyl group. When the molecule forms a stable conjugated structure at the end of the coupling reaction, one electron is lost, and the molecule becomes a cation skeleton. Therefore, polymethine dyes generally consist of a cationic skeleton and nonpolar anion, such as the boron tetrafluoride ion (BF_4_^−^) or perchlorate ion (ClO_4_^−^), as reviewed recently [53].

The optical absorption of polymethine dyes changes with solvent polarity [50, 54, 55]. This is because the polarity of the solvent changes the energy gap between the highest occupied molecular orbital (HOMO) and lowest unoccupied molecular orbital (LUMO). In low-polarity solvents, the charge of the polymethine moiety is delocalized (cyanine limit state), whereas the charge is localized in highly polar solvents, resulting in an increase in dipole moment [54] (Fig. 2b). For example, when IR-26 is dissolved in dichloromethane (low polarity), dimethyl sulfoxide (DMSO; medium polarity), and water (high polarity), the cyanine limit state and fluorescence intensity increase as the polarity of the solvent decreases [50]. Polymethine dyes are ionic molecules with cationic backbones and counter anions such as ClO_4_^−^ and BF_4_^−^ (Fig. 2a) [52]. These counter ions have a high molecular weight (>80 g/mol) and tetrahedral structure consisting of sp^3^ orbitals. The large anions are located away from the cation center of the backbone, which weakens the electrostatic forces between the ions and reduces the polarization of the entire molecule. The tetrahedral structure of the anion also cancels out the polarization between atoms, thus minimizing the polarity of the entire counter ion. The counter anion, which weakens the intramolecular polarization of the dye and has low polarity, inhibits thermal relaxation of the polymethine dye from its excited states, resulting in high NIR fluorescence properties. On the other hand, when the dye contacts water molecules, the counter anion is replaced by OH^−^, which has a low molecular weight (17 g/mol) and high polarity and thus induces thermal relaxation of the dye from its excited state [39, 53]. This quenching is also due to their small HOMO–LUMO gap, which corresponds to the lower photon energy of OTN-NIR light than that of visible and NIR-I light. Therefore, the contact of the dye with water should be suppressed to prevent quenching for the development of brightly emissive OTN-NIR fluorescent probes using dyes.

The absorption (excitation) wavelengths of the polymethine dyes were also changed by heterocyclic modification. For example, the absorption wavelength of polymethine dyes shifts to longer wavelengths although the quantum yield decreases when the heteroatom is changed from oxygen to another group 16 element, sulfur, selenium, or tellurium [52]. Based on this concept, Flav5 was developed as a new polymethine dye with high stability and quantum yield [40]. BTC1070 is also an OTN-NIR fluorophore developed by adding an electron-donating dimethylamino group to the benzothiopyrilium backbone [50]. The extension of the conjugated structure is also effective in yielding fluorescent dyes that operate in the long-wavelength (i.e., OTN) NIR wavelength region. Thus, the development of new dyes suitable for OTN-NIR fluorescence imaging has been investigated.

## Nanoparticles Encapsulating Near-Infrared Fluorescent Dye

**Fig. 3:**
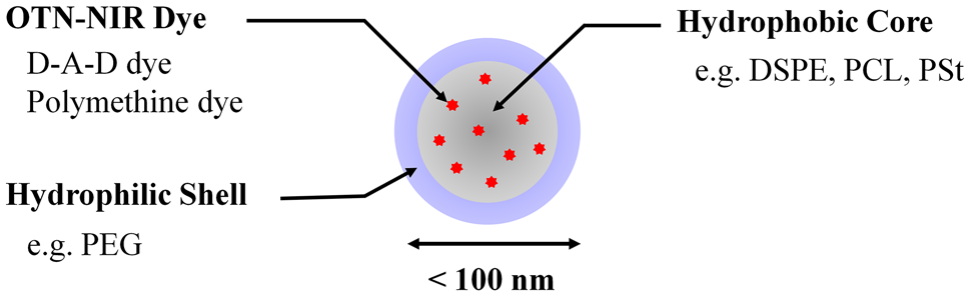
Schematic structure of OTN-NIR fluorescent nanoparticle with a hydrophobic core and hydrophilic shell. *DSPE* 1,2-distearoyl-sn-glycero-3-phosphoethanolamine, *NIR* near infrared, *OTN* over-thousand nanometers, *PCL* polycaprolactone, *PEG* poly(ethylene glycol); *PSt* polystyrene

The fluorescence of OTN-NIR dyes is significantly reduced when coupled with highly polar water molecules owing to their large phonon energy with polar vibrations [53]. Therefore, encapsulating the dye in the hydrophobic core of nanoparticles, which can shield the dyes from the effect of water molecules, is effective for their application in fluorescence imaging in aqueous and physiological environments. OTN-NIR fluorescence imaging using polymethine dyes was first reported as real-time angiography for the whole body of mice after intravenous injection of 6 nm diameter nanoparticles of an IR-1061 and polyacrylic acid mixture encapsulated in micelles of a phospholipid derivative containing poly(ethylene glycol) (PEG), 1,2-distearoyl-sn-glycero-3-phosphoethanolamine (DSPE)-PEG [56]. The structure of the micellar nanoparticles was presumed to be such that the stearoyl group, a fatty acid chain, forms a hydrophobic core, PEG forms a hydrophilic shell, and IR-1061 is encapsulated in the core. Subsequently, many OTN-NIR fluorescent micellar nanoparticles with polymethine dyes encapsulated in various hydrophobic cores, such as DSPE [57, 58], other lipids [59], polycaprolactone (PCL) [60], and polystyrene (PSt) [61] have been reported (Fig. 3). These studies considered encapsulating the dye into the hydrophobic core via hydrophobic interactions. Recently, rational methods have been reported to design a probe that can control dye encapsulation into the hydrophobic core and maximize the optical properties of the dye in nanoparticles. In a recent study of IR-1061 encapsulated in anionic, nonionic, and cationic phospholipids [62], the anionic phospholipid showed the best fluorescence performance, indicating that the charge is a key parameter in designing useful nanoparticles to encapsulate polymethine dyes. The characteristics of the hydrophobic part, where the dye interacts directly, also influences the performance of the obtained fluorescent probes. Investigating the relationship between the chemical properties of the hydrophobic core in the core–shell nanoparticles and the chemical and optical properties of the dye led to controlled dye encapsulation in the hydrophobic core.

## Screening Methods for Stability of Polymer Nanoparticles Using Environmentally Responsive Dye Molecules

For biomedical applications of polymer nanoparticles, it is useful to evaluate not only the particulate structural stability but also the encapsulation stability of functional molecules inside the core. Depending on the physicochemical properties of the polymer nanoparticles, water molecules in the dispersion medium may affect the hydrophobic core encapsulating molecules. For example, if a molecule is water-sensitive, a slight penetration of water may cause loss or change of its function, or aggregation of encapsulated molecules may cause the function to degenerate. The influence of water in the medium on the hydrophobic core can also be affected by the presence of ions or proteins. This is because their collisions with nanoparticle’s shell may affect the core stability, when the mobility of the core constituent molecules is responsive to forces from the environment.

**Fig. 4:**
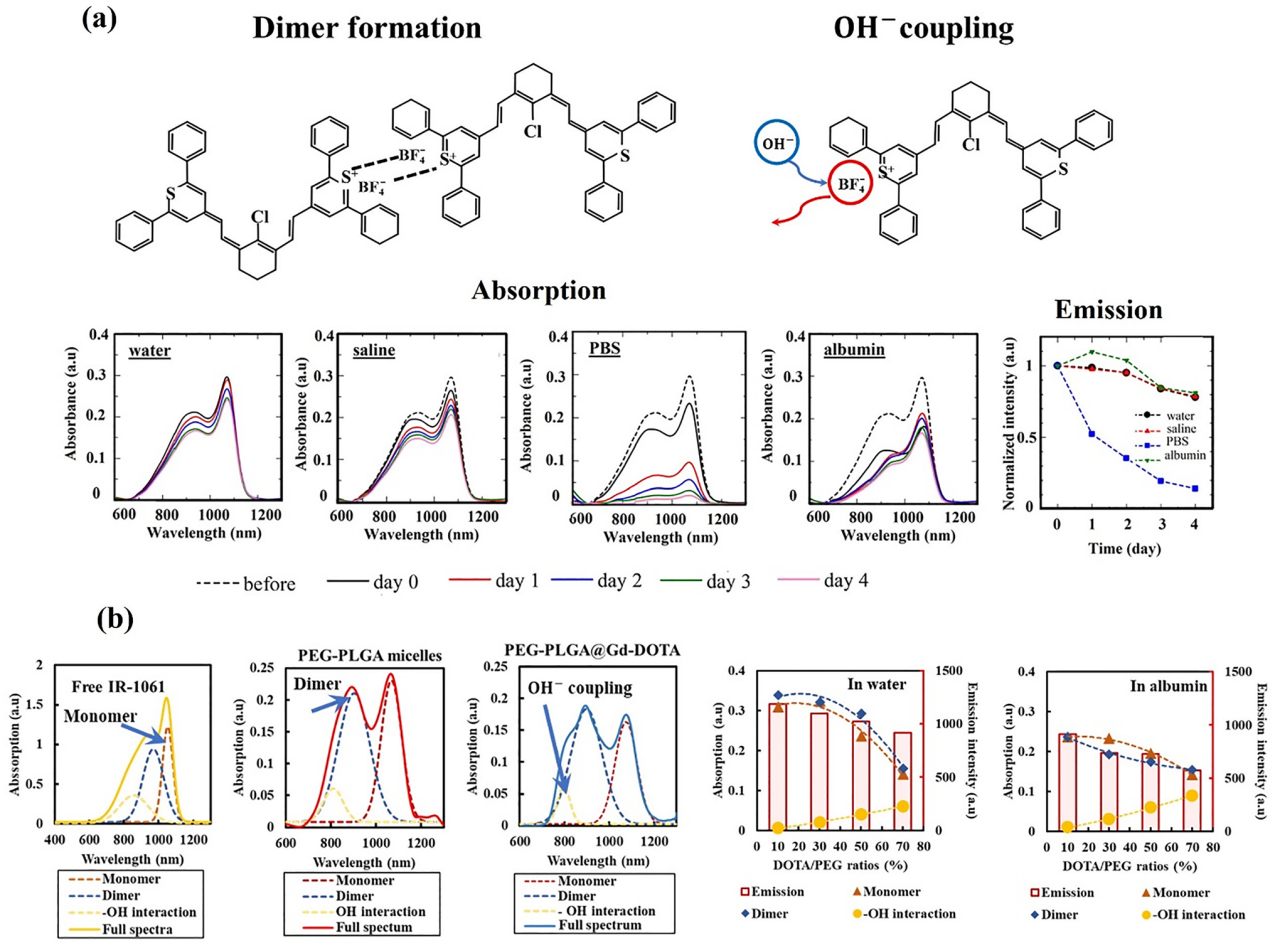
Effect of monomers, dimers, and OH^–^ coupling on the stability of nanostructures. **a** Mechanism of dimer and OH^–^ coupling formation and the absorption and emission of PEG-DSPE micelles were investigated in four different media for 4 days. **b** Deconvoluted absorption spectra of free IR-1061 in ACN, and IR-1061 encapsulated in PEG-*b*-PLGA and Gd-DOTA-conjugated PEG-*b*-PLGA micelles. The correlation between monomers, dimers, and OH^–^ coupling on fluorescence stability was determined in water and in albumin. Figures adapted with permission [66, 70]

Polymer nanoparticle models that encapsulate environmentally responsive molecules, such as dye molecules which change their optical properties under the influence of water, are useful for evaluating the stability of the hydrophobic core. By measuring the optical properties (e.g., absorption spectra) of nanoparticles encapsulating such dye molecules in various environments and conditions, the changes in the stability of the encapsulated molecules depending on the core constituents over time can be analyzed. For example, IR-1061, a polar and hydrophobic molecule consisting of two heterocyclic terminal moieties with one resonance positive charge connected by a polymethine bridge with a counter ion BF_4_^–^ [60, 63–65] (Fig. 4), is useful as a dye that is sensitive to a slight water. At low concentrations, these molecules are distributed as monomers. When the concentration is increased, a dimer distribution is formed owing to the attraction between the positive terminal of one molecule and the BF_4_^–^ center of the other [58, 66]. IR-1061 also interacts with the hydroxyl ions (OH^–^) of surrounding water molecules (Fig. 4a). As a hydrophobic dye, IR-1061 can be encapsulated in the hydrophobic core of polymeric nanoparticles with the chemical composition described previously [56, 60, 61, 67]; however, the BF_4_^–^ counter anion is easily replaced by OH^–^ upon the approach of water molecules present in the surrounding hydrophilic environment, resulting in a significant change in absorption wavelength [68]. When encapsulated inside a micelle core, dimer formation is attributed to the Hansen solubility parameter (HSP) affinity between the dye and hydrophobic core [66, 67, 69]. The matching of HSP affinities results in monomer distribution, whereas mismatching causes dimer formation [58, 67].

The monomer, dimer, and OH^–^ coupling distributions are critical when using micelles in vivo; multiple interactions between the micelles and blood components result in micelle instability and fluorescence quenching. This dye’s property of responsive to the microenvironment can be applied for a simple in vitro screening method, as introduced by Doan and Umezawa, to identify the factors that negatively affect stability [66]. This model evaluated the stability of IR-1061 encapsulated in PEG-DSPE micelles by interpreting the correlation between monomers, dimers, and OH^–^ coupling versus the fluorescence intensity and diameters of the micelles dispersed in four types of media, including water, physiological saline, phosphate-buffered saline (PBS), and aqueous albumin solution. Saline is used to assess the impact of isotonic concentrations of salts (ions), while PBS and albumin solution are for the impacts of coexistence of high-valence ions and bio-macromolecules, respectively. The results indicated a mismatch in affinity between the DSPE core and IR-1061, which rapidly caused structural instability and fluorescence quenching in PBS and albumin.

This method was successfully applied to investigate the structural stability of IR-1061-encapsulated micelles composed of gadolinium(III) 1,4,7,10-tetraazacyclododecane-1,4,7,10-tetraacetate (Gd-DOTA) conjugated to poly(ethylene glycol) methyl ether-*block*-poly(lactide-*co*-glycolide) (PEG-*b*-PLGA) using a combination of OTN-NIR and MRI. The structure of Gd-DOTA contains multiple carboxyl groups, making it highly hydrophilic and attracting water molecules. When conjugated into PEG-*b*-PLGA micelles, the conjugation induces OH^–^ coupling of IR-1061, resulting in reduced stability and quenching. In these studies, the stability was improved by differentiating the Gd-DOTA/PEG-*b*-PLGA ratios [71] and the Gd-DOTA locations [70] as evaluated by interpreting the correlation between monomers, dimers, and OH^–^ coupling factors with the fluorescence intensity. The results showed that increasing the dimer distribution and OH^–^ coupling reduced fluorescence. By contrast, reducing the dimer distribution led to an increase in the fluorescence intensity, while reducing OH^–^ coupling improved the structural stability [70, 71]. The absorption spectra of IR-1061 in acetonitrile (ACN) and inside the cores of PEG-*b*-PLGA and Gd-DOTA-conjugated PEG-*b*-PLGA micelles attributed to monomers, dimers, and/or the OH^–^ coupling effect between IR-1061 and the OH^–^ in water are shown in Fig. 4b. The change in the optical property of IR-1061 depending on its dimer formation is similar to that of aggregate formation of ICG [72]. The figure illustrates the effect of the Gd-DOTA ratio conjugated to PEG-*b*-PLGA micelles on the correlation between monomers, dimers, and OH^–^ coupling with fluorescence intensity in water and albumin.

## Effect of the Polarity of the Polystyrene Core on Encapsulation of OTN-NIR Fluorescent Dyes

Nanoparticles with hydrophobic cores can be synthesized by self-assembly in a solution of amphiphilic molecules or through the polymerization of monomers. In the method where the hydrophobic core is formed by self-assembly, micellar and lipid nanoparticles can be obtained [2, 4, 73, 74] as nanoparticles with hydrophobic cores that are easily degraded by physicochemical stimuli. By contrast, hydrophobic cores synthesized via radical polymerization (with molecular weights of ca. 5000−50000) are stable under physiological conditions. For example, PSt particles are stable in plasma and are widely used as in vitro diagnostic agents for latex agglutination immunoturbidimetry [75, 76].

**Fig. 5:**
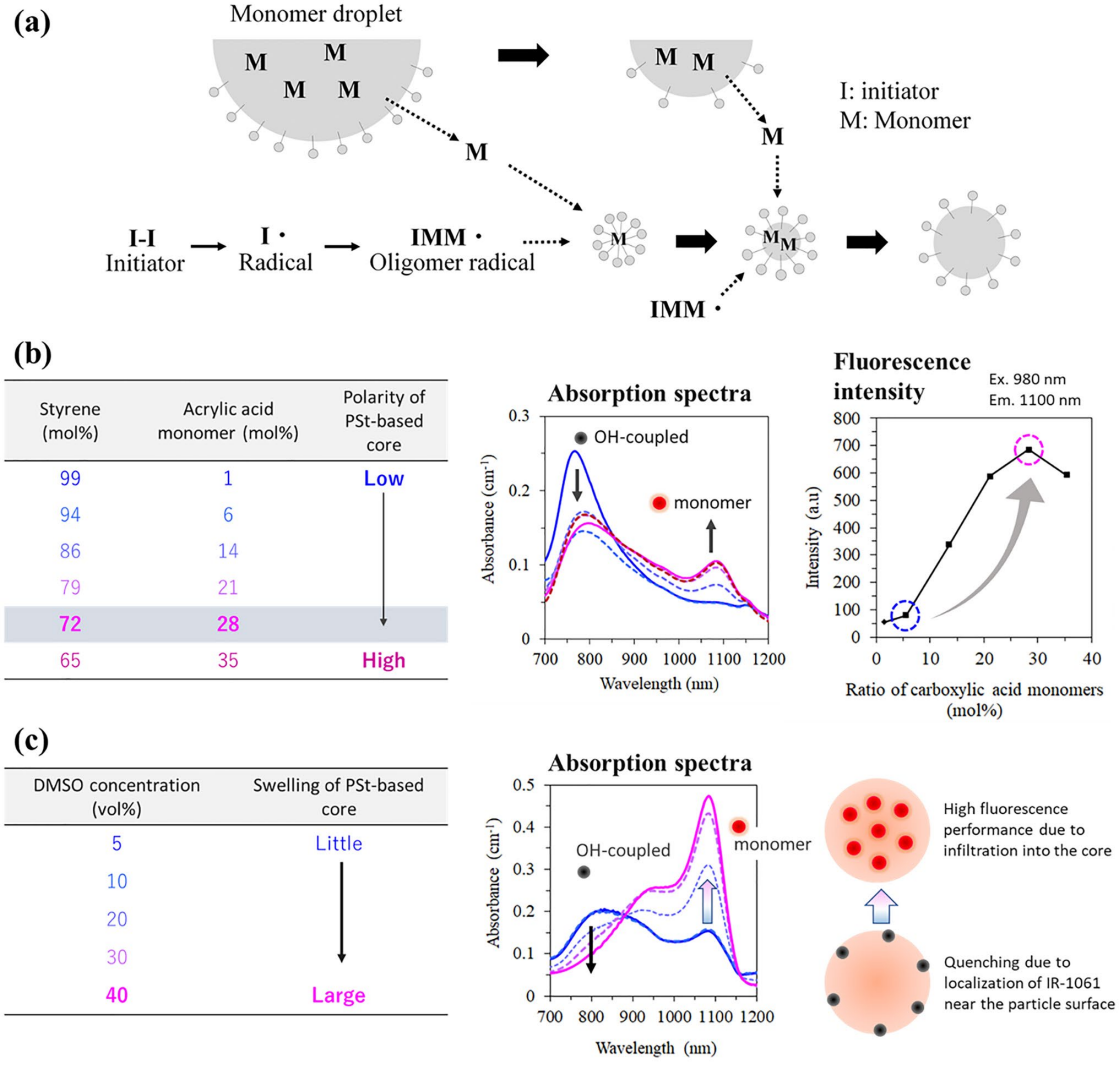
Synthesis scheme of PSt nanoparticles with IR-1061, an OTN-NIR fluorescent dye, to yield high fluorescence. **a** Schematic mechanism of emulsion polymerization to obtain polymer nanoparticles that can encapsulate OTN-NIR dye molecules. **b** Effect of introducing polar (carboxy) groups into hydrophobic polymer cores on the encapsulation of OTN-NIR dyes. **c** Effect of adding an organic solvent (DMSO) to enhance the swelling of PSt nanoparticles to allow infiltration of the dye into the nanoparticle core [68]

Radical polymerization includes emulsion polymerization, dispersion polymerization, precipitation polymerization, and suspension polymerization, which produce different ranges of particle sizes [77, 78]. The in vivo behavior of nanoparticles is greatly influenced by the particle size; they are excreted via the kidney to the urine if the particle size is <5 nm [79], and remain trapped in the reticuloendothelial system (mainly the liver and spleen) if the particle size is >200 nm. Nanoparticles of 10–100 nm in diameter, which can be synthesized by emulsion polymerization, are preferable because this size range allows control over their in vivo behavior with high blood retention and potential tumor invasiveness [80]. In this process, emulsified monomers are polymerized in the aqueous phase, in which the surfactants and polymerization initiators are dissolved. Oligomeric radicals generated by the initiator enter the hydrophobic core of micelles containing monomers, where they repeatedly react to form polymerized nanoparticles (Fig. 5a). The composition of the core can be tuned by varying the monomer used for polymerization, which allows the relationship between the chemical properties of the hydrophobic core and optical properties of the encapsulated dyes to be investigated [68].

Styrene is a hydrophobic monomer of low polarity [81], whereas acrylic acid is a water-soluble and highly polar monomer with carboxyl groups. Therefore, the polarity of the PSt core can be controlled by changing the ratio of acrylic acid introduced into PSt. IR-1061 can be introduced into the PSt-based solid nanoparticles (diameter: ≈ 50 nm) via the swelling-diffusion method [82] during emulsion polymerization. The amount of encapsulated OTN-NIR fluorescent IR-1061 dye and the fluorescence performance of the resulting dye-encapsulated PSt-based nanoparticles were positively correlated with the acrylic acid ratio (≈ 30 mol% in PSt), that is, the polarity of the PSt-based particle core [68] (Fig. 5b). This is because IR-1061 is hydrophobic yet polar and ionized, with a low affinity for PSt owing to the high hydrophobicity of this polymer. Adding acrylic acid units to PSt increased the polarity of the PSt-based nanoparticle core and the ability to encapsulate IR-1061 by improving their affinity. In addition, by increasing the DMSO concentration (≈ 40 vol%) to swell the PSt nanoparticles and introduce the IR-1061 dye, the fluorescence intensity of the resulting PSt nanoparticles further increased as the dye infiltrated the interior of the hydrophobic PSt-based core, reducing contact with water molecules in the dispersion medium (Fig. 5c). A hydrophilic shell layer is needed for dispersibility in a physiological environment. This is obtained by chemically modifying the acrylic acid unit in the polymer comprising the nanoparticle core via covalently attaching PEG to an oligoamine at one end. This OTN-NIR fluorescent dye-loaded PEGylated PSt-based nanoparticles showed high dispersion stability under physiological conditions in the presence of serum proteins. The designed OTN-NIR fluorescent PSt-based nanoparticles can also be used for angiography several hours after intravenous injection in mice [68]. Thus, adjusting the polarity of the hydrophobic polymer core using NIR fluorescent dye molecules is useful for designing NIR fluorescent probes comprised of polymer nanoparticles.

## Effect of the Polarity of the Hydrophobic Core of Micellar Nanoparticles on the Encapsulation of OTN-NIR Fluorescent Dyes

The polarity of the hydrophobic core of the nanoparticles influences dye encapsulation into the core, as described above. A quantitative method to evaluate the polarity of the hydrophobic core and dye molecules by controlling their affinity will lead to the efficient design of nanoparticle probes emitting OTN-NIR fluorescence. There is a solubility parameter (SP), which is defined as the proportion of cohesive energy density as proposed in 1949 [83], that can provide a measure of their affinity between two molecules. SP is a thermodynamic parameter for liquids, but it can be applied to discuss the solubility of solids in a solvent [84–86] as well as the compatibility of two solids, such as polymers and carbon nanomaterials [87], or polymer nanoparticles and epoxy resin [88]. Thus, SP can be applied to organic molecules by examining the solubility and turbidity of polymers and powders in solvents with different SP values [89]. Among several SPs, the Hansen solubility parameter (HSP) is described by three components: dispersion, polarity, and hydrogen bonding [90, 91]. While the HSP helps to estimate miscibility, adhesion, and wetting, it is mainly used to guide organic solvent selection, salt screening, and solid dispersion in the pharmaceutical field [92]. This parameter is also effective for evaluating the affinity between polymer and dye molecules, allowing control over the encapsulation of dyes into polymeric micellar nanoparticles [67].

**Fig. 6:**
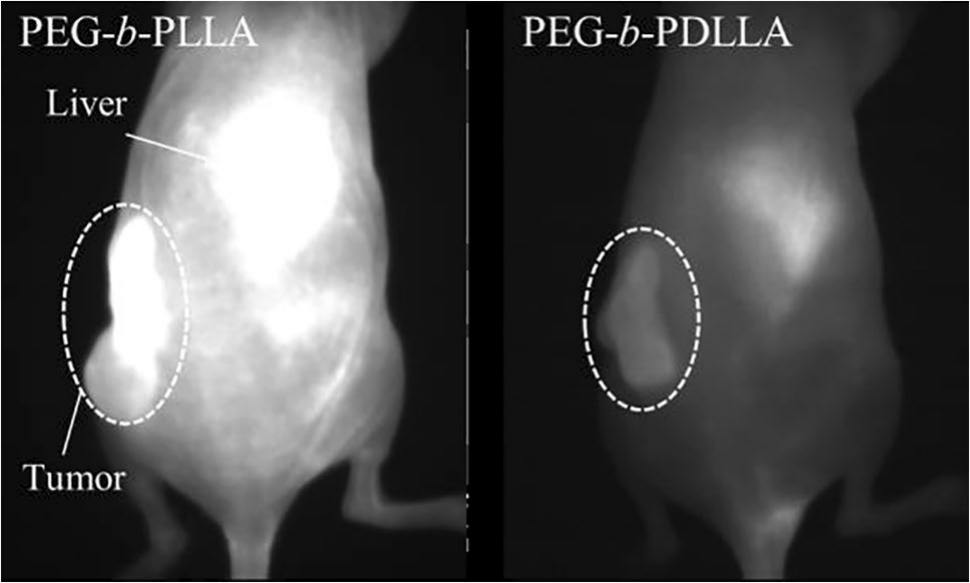
Difference in the retention of in vivo OTN-NIR fluorescence (excitation at 980 nm, emission at 1100 nm) of IR-1061-loaded PEG-*b*-PLA micelles with different chiral structures of the PLA core: PLLA, and PDLLA. The physicochemical stability of these micelles were reported in previous studies [108].The images were acquired at 24 h after intravenous injection of the fluorescent micelles in mice with a subcutaneous tumor model

In addition, there is a method for calculating the HSP values of molecules. This method assumes that the HSP values depend on the number and type of functional groups, and the molecular structure is divided into accordingly. Although the HSP values of ionic molecules, including polymethine dyes, cannot be calculated directly from their molecular structures based on this method [93], they can be obtained from the results of dissolution tests of the target substance in various solvents with known HSPs [94, 95]. PLGA [96, 97], poly(lactic acid) (PLA) [98–100], PCL [60, 98, 101, 102], PSt [61, 102, 103], and phospholipid such as DSPE [57, 104, 105] consists of hydrophobic cores of micellar nanoparticles encapsulating hydrophobic polymethine dyes. Fluorescence micelles of polymers can be prepared by encapsulating the polymethine dye IR-1061 in hydrophobic cores via co-solvent evaporation [106]. Fluorescent micellar nanoparticles with a polymer core (PLGA and PLA) that had a chemical structure with a small energy difference [107] from IR-1061 showed stable and highly effective encapsulation of the dye and thus high fluorescence performance [67] (Fig. 6). In particular, PCL and DSPE, which have been widely reported as a component enabling the encapsulation of IR-1061 were found to be poorly compatible with IR-1061. In fact, the absorption spectra of the micellar nanoparticles encapsulating IR-1061 contained a peak at ≈ 780 nm owing to the influence of water molecules on the dye in the case of DSPE [57, 62] and PCL [60] hydrophobic cores. Note that the increased affinity between the dye and polymer prevents the invasion of water molecules, which can couple with IR-1061 as a quencher, into the hydrophobic core of the polymer micelles. Suppressing the invasion of water molecules improves the stability of fluorescent polymer micelles in aqueous and physiological environments.

## Effect of the Molecular Weight of the Hydrophobic Core on Fluorescence Performance of Micellar Nanoparticles Encapsulating OTN-NIR Dyes

The encapsulation efficiency and stability of small molecules in micellar nanoparticles depend on not only the chemical structure of the core polymer but also the molecular weight of the polymer that forms the core. For example, the effects of the polymer chain length of PEG-*b*-PCL on the stability and biocompatibility of micellar nanoparticles have been reported [109]. Investigating the effect of the molecular weight of the hydrophobic core polymer, which can also affect dye encapsulation in the core, is important for designing high-performance OTN-NIR fluorescent-dye-encapsulated nanoparticles.

For IR-1061-encapsulated micelles with shells composed of PEG, a biocompatible hydrophilic polymer, the effect of the molecular weight of a PLGA core polymer, which has a high affinity for the dye, was investigated [110]. In this study, the fluorescence performances of micellar nanoparticles encapsulating IR-1061 in PEG-*b*-PLGA with PLGA molecular weights of 1000, 2000, and 5000, and a PEG molecular weight of 2000 were compared. Within this range of PLGA molecular weights (i.e., 1000–5000), the higher the molecular weight of the PLGA core, the higher the dye encapsulation efficiency and fluorescence intensity. Their absorption spectra showed that IR-1061 encapsulated in high-molecular-weight PLGA core was less affected by water molecules with a large phonon energy of polar vibration of hydroxyl bonding [53] in the dispersant [110]. The IR-1061-encapsulated PEG-*b*-PLGA micelles were highly stable in both in vitro (PBS containing albumin) and in vivo (in the circulating blood of mice) environments, as evidenced by the increase in the molecular weight of PLGA constituting the micelle core [110]. Their time-dependent decrease in fluorescence intensity in these physiological environments was attributed to micelle deformation by proteins and salts, suggesting that the molecular weight of the hydrophobic polymer core is involved in the robustness of micelles encapsulating the polymethine dye IR-1061.

## Effect of Chiral Structure of Poly(Lactic Acid) in the Core on In Vivo Stability of OTN-NIR Dye-Loaded Micellar Nanoparticles

In addition to polarity and molecular weight, the homogeneity of the chiral structure of the hydrophobic core polymer of micellar nanoparticles with encapsulated IR-1061 dye also influences the in vivo imaging performance. In the field of DDS, PLA with different chiral structures, poly(L-lactic acid) (PLLA; pure L-form) and poly(D,L-lactic acid) (PDLLA; a mixture of D- and L-forms), have been reported to exhibit different encapsulation and release properties for small-molecule drugs [111, 112]. When loaded with ICG, the stereochemistry of the PLA core has been suggested to affect physical stability in the blood and tumor/background contrast in imaging [111]. The difference in the encapsulation properties of IR-1061 depending on the chiral structure of PLA was investigated by comparing IR-1061-loaded micellar nanoparticles synthesized using two types of PEG-*b*-PLA: PEG-*b*-PLLA and PEG-*b*-PDLLA. IR-1061 was more stable when encapsulated in the hydrophobic PLLA core of PLLA in micellar nanoparticles than when encapsulated in the PDLLA core. The PEG-*b*-PLLA micelles encapsulating IR-1061 showed higher fluorescence retention in in vivo tissues than PEG-*b*-PDLLA micelles encapsulating the dye, suggesting higher in vivo stability of PEG-*b*-PLLA (Fig. 6) [108]. Since PLLA has a higher crystallinity than PDLLA [113, 114], the stability is considered to be caused by the robustness of the core polymer. Thus, the in vivo stability of dyes in polymeric micelles is also affected by the robustness of their polymer cores, and the choice of hydrophobic-core polymers with high robustness is useful in designing probes that maintain a high fluorescence intensity.

## Conclusion and Perspectives

In this paper, the strategy of controlling the encapsulation of OTN-NIR fluorescent dyes in the hydrophobic core of core–shell nanoparticles was reviewed. The polarity and molecular weight of the hydrophobic core polymer influence the efficiency (amount) of the OTN-NIR dyes, fluorescence intensity, and stability of the obtained micellar nanoparticles. The polarity of the polymer core has a significant impact on the properties of nanoparticles encapsulating functional molecules and can be quantitatively evaluated in terms of HSP for various molecules. A quantitative evaluation of the polarity between the hydrophobic core polymer and the dye molecules using solubility parameters is useful for selecting a hydrophobic core polymer with a polarity matching that of the dye. Dye-encapsulated nanoparticles with optimized encapsulation and stability have been demonstrated to be applicable as OTN-NIR fluorescent contrast agents for vascular and tumor imaging in vivo.

For in vivo imaging, novel fluorescent dyes have been developed to improve the quantum yield and permeability of light by increasing the wavelengths of absorption and fluorescence from the visible range. The approach to control dye encapsulation in the hydrophobic core of nanoparticles is expected to enhance the imaging performance of these novel fluorescent dyes in physiological environments. The design concept presented in this article is expected to be utilized for mechanistic probe design considering chemical properties such as the molecular weight of the polymer and the polarities of dye and the hydrophobic core of the micelle. Although we focused on fluorescent dyes and NIR fluorescence imaging as encapsulation molecules and their application fields in this paper, our concept can be extended to other research fields and industries. For example, in drug-delivery systems, in which drug molecules are encapsulated in a hydrophobic core, nanoparticles are key materials. As discussed in this paper, controlling the encapsulation may also be effective in controlling the spatiotemporal in vivo dynamics of drugs in human and animal tissues. Biomolecules such as proteins and nucleic acids are also targeted as encapsulated molecules; a prime example is nucleic acids for pharmaceuticals and vaccines, in which siRNA or mRNA are encapsulated in lipid nanoparticles. In this case, RNA is encapsulated in a core composed of composite materials such as phospholipids, cationic lipids, PEG-modified lipids, and cholesterol. Since RNA is negatively charged, its polarity inside the core must be controlled. Based on the knowledge presented in this paper, it is expected that methodologies for more effective applications of various biomolecules and macromolecules with complex high-dimensional structures will be further established.

## References

[1] Riediker M (2019). Particle toxicology and health—where are we?. Part. Fibre Toxicol..

[2] Mitchell MJ (2021). Engineering precision nanoparticles for drug delivery. Nat. Rev. Drug Deliv..

[3] Ahmed Z (2014). Polymeric micelles as drug delivery vehicles. RSC Adv..

[4] Din FU (2017). Effective use of nanocarriers as drug delivery systems for the treatment of selected tumors. Int. J. Nanomed..

[5] Hamaguchi T (2005). NK105, a paclitaxel-incorporating micellar nanoparticle formulation, can extend in vivo antitumour activity and reduce the neurotoxicity of paclitaxel. Br. J. Cancer.

[6] Fujiwara Y (2019). A multi-national, randomised, open-label, parallel, phase III non-inferiority study comparing NK105 and paclitaxel in metastatic or recurrent breast cancer patients. Br. J. Cancer.

[7] Hou X (2021). Lipid nanoparticles for mRNA delivery. Nat. Rev. Mater..

[8] Schoenmaker L (2021). mRNA-lipid nanoparticle COVID-19 vaccines: Structure and stability. Int. J. Pharm..

[9] Reisch A, Klymchenko AS (2016). Fluorescent polymer nanoparticles based on dyes: seeking brighter tools for bioimaging. Small.

[10] Lim EK (2015). Nanomaterials for theranostics: recent advances and future challenges. Chem. Rev..

[11] Ghosh B, Biswas S (2021). Polymeric micelles in cancer therapy: state of the art. J. Control. Release.

[12] Cao Y (2014). Dual drug release from core–shell nanoparticles with distinct release profiles. J. Pharm. Sci..

[13] Baghbanbashi M, Kakkar A (2022). Polymersomes: soft nanoparticles from miktoarm stars for applications in drug delivery. Mol. Pharm..

[14] Weissleder R, Pittet MJ (2008). Imaging in the era of molecular oncology. Nature.

[15] Andreasen NC (1988). Evaluation of brain imaging techniques in mental illness. Ann. Rev. Med..

[16] Beckh S (2002). Real-time chest ultrasonography: a comprehensive review for the pulmonologist. Chest.

[17] Hong G (2017). Near-infrared fluorophores for biomedical imaging. Nat. Biomed. Eng..

[18] Soga K (2021). Transparency in Biology: Making Invisible Visible.

[19] Troyan SL (2009). The FLARE intraoperative near-infrared fluorescence imaging system: a first-in-human clinical trial in breast cancer sentinel lymph node mapping. Ann. Surg. Oncol..

[20] Tummers QR (2015). Intraoperative guidance in parathyroid surgery using near-infrared fluorescence imaging and low-dose Methylene Blue. Surgery.

[21] Anderson RR, Parrish JA (1981). The optics of human skin. J. Investig. Dermatol..

[22] Smith AM (2009). Second window for *in vivo* imaging. Nat. Nanotechnol..

[23] Welsher K (2011). Deep-tissue anatomical imaging of mice using carbon nanotube fluorophores in the second near-infrared window. Proc. Natl. Acad. Soc. USA.

[24] Jaque D (2016). Inorganic nanoparticles for optical bioimaging. Adv. Opt. Photon..

[25] Umezawa M (2020). Computed tomography for in vivo deep over-1000 nm near-infrared fluorescence imaging. J. Biophoton..

[26] Soga K (2019). Near-infrared Biomedical Imaging for Transparency. J. Imaging Soc. Jpn..

[27] Welsher K (2009). A route to brightly fluorescent carbon nanotubes for near-infrared imaging in mice. Nat. Nanotechnol..

[28] Iizumi Y (2018). Oxygen-doped carbon nanotubes for near-infrared fluorescent labels and imaging probes. Sci. Rep..

[29] Takeuchi T (2019). Characterization and biodistribution analysis of oxygen-doped single-walled carbon nanotubes used as in vivo fluorescence imaging probes. Bioconjug. Chem..

[30] Sekiyama S (2019). Delayed increase in near-infrared fluorescence in cultured murine cancer cells labeled with oxygen-doped single-walled carbon nanotubes. Langmuir.

[31] Hong G (2012). In vivo fluorescence imaging with Ag2S quantum dots in the second near-infrared region. Angew. Chem. Int. Ed..

[32] Nakane Y (2013). Aqueous synthesis of glutathione-coated PbS quantum dots with tunable emission for non-invasive fluorescence imaging in the second near-infrared biological window (1000–1400 nm). Chem. Commun..

[33] Soga K (2010). Application of ceramic phosphors for near infrared biomedical imaging technologies. Proc. SPIE.

[34] Naczynski DJ (2013). Rare-earth-doped biological composites as in vivo shortwave infrared reporters. Nat. Commun..

[35] Hemmer E (2013). Upconverting and NIR emitting rare earth based nanostructures for NIR-bioimaging. Nanoscale.

[36] Kamimura M (2017). Ratiometric near-infrared fluorescence nanothermometry in the OTN-NIR (NIR II/III) biological window based on rare-earth doped β-NaYF_4_ nanoparticles. J. Mater. Chem. B.

[37] Ortgies DH (2018). Lifetime-encoded infrared-emitting nanoparticles for in vivo multiplexed imaging. ACS Nano.

[38] Chihara T (2019). Biological deep temperature imaging with fluorescence lifetime of rare-earth-doped ceramics particles in the second NIR biological window. Sci. Rep..

[39] Okubo K (2021). Concept and application of thermal phenomena at 4f electrons of trivalent lanthanide ions in organic/inorganic hybrid nanostructure. ECS J. Solid State Sci. Technol..

[40] Cosco ED (2017). Flavylium polymethine fluorophores for near- and shortwave infrared imaging. Angew. Chem. Int. Ed..

[41] Li B (2018). An efficient 1064 nm NIR-II excitation fluorescent molecular dye for deep-tissue high-resolution dynamic bioimaging. Angew. Chem. Int. Ed..

[42] Meng X (2018). Hypoxia-triggered single molecule probe for high-contrast NIR II/PA tumor imaging and robust photothermal therapy. Theranostics.

[43] Yi W (2019). A NIR-II fluorescent probe for articular cartilage degeneration imaging and osteoarthritis detection. Biomater. Sci..

[44] Bachilo SM (2002). Structure-Assigned Optical Spectra of Single-Walled Carbon Nanotubes. Science.

[45] Onoda A, Umezawa M, Soga K (2021). Carbon nanotubes—potential of use for deep bioimaging. Transparency in Biology: Making Invisible Visible.

[46] Tezuka K (2021). Upconversion luminescent nanostructure with ultrasmall ceramic nanoparticles coupled with rose bengal for NIR-induced photodynamic therapy. ACS Appl. Bio Mater..

[47] Ding F (2018). Recent advances in near-infrared II fluorophores for multifunctional biomedical imaging. Chem. Sci..

[48] Li L (2020). A short review on NIR-II organic small molecule dyes. Dyes Pigments.

[49] Lei Z, Zhang F (2021). Molecular engineering of NIR-II fluorophores for improved biomedical detection. Angew. Chem. Int. Ed..

[50] Wang S (2019). Anti-quenching NIR-II molecular fluorophores for in vivo high-contrast imaging and pH sensing. Nat. Commun..

[51] Bricks JL (2015). Molecular design of near infrared polymethine dyes: a review. Dyes Pigm..

[52] Detty MR, Murray BJ (1982). Telluropyrylium dyes. 1. 2,6-Diphenyltelluropyrylium dyes. J. Org. Chem..

[53] Okubo K (2021). Near infrared fluorescent nanostructure design for organic/inorganic hybrid system. Biomedicines.

[54] Bouit PA (2010). Continuous symmetry breaking induced by ion pairing effect in heptamethine cyanine dyes: beyond the cyanine limit. J. Am. Chem. Soc..

[55] Pascal S (2014). Expanding the polymethine paradigm: evidence for the contribution of a bis-dipolar electronic structure. J. Phys. Chem. A.

[56] Tao Z (2013). Biological imaging using nanoparticles of small organic molecules with fluorescence emission at wavelengths longer than 1000 nm. Angew. Chem. Int. Ed..

[57] Chen Q (2019). Novel small molecular dye-loaded lipid nanoparticles with efficient near-infrared-II absorption for photoacoustic imaging and photothermal therapy of hepatocellular carcinoma. Biomater. Sci..

[58] Umezawa M (2022). Heat treatment effects for controlling dye molecular states in hydrophobic core of over-1000nm near-infrared (NIR-II) fluorescent micellar nanoparticles. ACS Omega.

[59] Xie X (2019). A targeted biocompatible organic nanoprobe for photoacoustic and near-infrared-II fluorescence imaging in living mice. RSC Adv..

[60] Kamimura M (2017). Over-1000 nm near-infrared fluorescent biodegradable polymer nanoparticles for deep tissue in vivo imaging in the second biological window. Polym. J..

[61] Umezawa M (2021). Effects of processing pH on emission intensity of over-1000 nm near-infrared fluorescence of dye-loaded polymer micelle with polystyrene core. Anal. Sci..

[62] Yu H (2021). Rational design of a NIR-II fluorescent nanosystem with maximized fluorescence performance and applications. Mater. Adv..

[63] Dung DKT (2020). Development of molecular imaging probe for dual NIR/MR imaging. J. Photopolym. Sci. Technol..

[64] Casalboni M (2003). Fluorescence efficiency of four infrared polymethine dyes. Chem. Phys. Lett..

[65] Ishchenko AA (1992). Effect of the polymethine chain length on the fluorescence spectrum of symmetric cyanine dyes. Opt. Spectrosc..

[66] Doan TKD (2022). Influence of physiological media on over-1000 nm NIR fluorescent DSPE-PEG micelles for bio-imaging. Chem. Lett..

[67] Ueya Y (2021). Design of over-1000 nm near-infrared fluorescent polymeric micellar nanoparticles by matching the solubility parameter of the core polymer and dye. ACS Nanosci. Au.

[68] Ueya Y (2021). Designing highly emissive over-1000-nm near-infrared fluorescent dye-loaded polystyrene-based nanoparticles for in vivo deep imaging. RSC Adv..

[69] Rüttger F (2019). Isomerization and dimerization of indocyanine green and a related heptamethine dye. Eur. J. Org. Chem..

[70] Doan TKD (2022). The effect of Gd-DOTA locations within PLGA-*b*-PEG micelles encapsulated IR-1061 on bimodal over-1000 nm near-infrared fluorescence and magnetic resonance imaging. Biomater. Sci..

[71] Doan TKD (2022). The influence of Gd-DOTA conjugating ratios to PLGA-PEG micelles encapsulated IR-1061 on bimodal over-1000 nm near-infrared fluorescence and magnetic resonance imaging. Biomater. Sci..

[72] Ji C (2021). Organic dye assemblies with aggregation-induced photophysical changes and their bio-applications. Aggregate.

[73] Szczęch M, Szczepanowicz K (2020). Polymeric core-shell nanoparticles prepared by spontaneous emulsification solvent evaporation and functionalized by the layer-by-layer method. Nanomaterials.

[74] Dai H (2021). NIR-II Organic nanotheranostics for precision oncotherapy. Small.

[75] Sarikaputi M (1992). Latex agglutination test: a simple, rapid and practical method for bovine serum CRP determination. Jpn. J. Vet. Res..

[76] Polpanich D (2007). Detection of malaria infection via latex agglutination assay. Anal. Chem..

[77] Arshady R (1992). Suspension, emulsion, and dispersion polymerization: a methodological survey. Colloid Polym. Sci..

[78] Lovell PA, Schork FJ (2020). Fundamentals of emulsion polymerization. Biomacromol.

[79] Choi HS (2010). Rapid translocation of nanoparticles from the lung airspaces to the body. Nat. Biotechnol..

[80] Cabral H (2011). Accumulation of sub-100 nm polymeric micelles in poorly permeable tumours depends on size. Nat. Nanotechnol..

[81] Lane WH (1946). Determination of Solubility of Styrene in Water and of Water in Styrene. Ind. Eng. Chem. Anal. Ed..

[82] Lee JH (2011). Dye-labeled polystyrene latex microspheres prepared via a combined swelling-diffusion technique. J. Colloid Interface Sci..

[83] Hildebrand J, Scott R (1949). The Solubility of Non-electrolytes.

[84] Jouyban A (2008). Review of the cosolvency models for predicting solubility of drugs in water-cosolvent mixtures. J. Pharm. Pharmacol..

[85] Nakano H, Nakamura D (2019). Hansen solubility parameters of stacked silicanes derived from porous silicon. ACS Omega.

[86] Easley A (2020). Nitroxide radical polymer–solvent interactions and solubility parameter determination. Macromolecules.

[87] Ma J, Larsen RM (2013). Comparative study on dispersion and interfacial properties of single walled carbon nanotube/polymer composites using Hansen solubility parameters. ACS Appl. Mater. Interfaces.

[88] Ning N (2021). Building effective core/shell polymer nanoparticles for epoxy composite toughening based on Hansen solubility parameters. Nanotechnol. Rev..

[89] Tsutsumi S (2019). Determination of Hansen solubility parameters of particles using a capillary penetration method. Chem. Phys..

[90] Hansen CM (1967). The Three Dimensional Solubility Parameter and Solvent Diffusion Coefficient, Their Importance in Surface Coating Formulation.

[91] Hansen CM (2007). Hansen Solubility Parameters A User's Handbook.

[92] Jankovic S (2019). Application of the solubility parameter concept to assist with oral delivery of poorly water-soluble drugs—a PEARRL review. J. Pharm. Pharmacol..

[93] Fujiwara N (2019). Evaluation of the influence of fine particle surface modification with the Hansen solubility parameters. Mater. Chem. Phys..

[94] Hansen CM, Smith AL (2004). Using Hansen solubility parameters to correlate solubility of C_60_ fullerene in organic solvents and in polymers. Carbon.

[95] Wieneke JU (2012). Systematic investigation of dispersions of unmodified inorganic nanoparticles in organic solvents with focus on the Hansen solubility parameters. Ind. Eng. Chem. Res..

[96] Makadia HK, Siegel SJ (2011). Poly lactic-co-glycolic acid (PLGA) as biodegradable controlled drug delivery carrier. Polymers.

[97] Zhang K (2014). PEG-PLGA copolymers: their structure and structure-influenced drug delivery applications. J. Control. Release.

[98] Ahmed F, Discher DE (2004). Self-porating polymersomes of PEG-PLA and PEG-PCL: hydrolysis-triggered controlled release vesicles. J. Control. Release.

[99] Lassalle V, Ferreira ML (2007). PLA nano- and microparticles for drug delivery: an overview of the methods of preparation. Macromol. Biosci..

[100] Xiao RZ (2010). Recent advances in PEG-PLA block copolymer nanoparticles. Int. J. Nanomed..

[101] Grossen P (2017). PEG-PCL-based nanomedicines: a biodegradable drug delivery system and its application. J. Control. Release.

[102] Kubát P (2020). Polystyrene and poly(ethylene glycol)- b-poly(ε-caprolactone) nanoparticles with porphyrins: structure, size, and photooxidation properties. Langmuir.

[103] Bhargava P (2006). Self-assembled polystyrene-block-poly(ethylene oxide) micelle morphologies in solution. Macromolecules.

[104] Wang R (2012). Application of poly(ethylene glycol)–distearoylphosphatidylethanolamine (PEG-DSPE) block copolymers and their derivatives as nanomaterials in drug delivery. Int. J. Nanomed..

[105] Che J (2015). DSPE-PEG: a distinctive component in drug delivery system. Curr. Pharm. Des..

[106] Aliabadi HM (2007). Encapsulation of hydrophobic drugs in polymeric micelles through co-solvent evaporation: the effect of solvent composition on micellar properties and drug loading. Int. J. Pharm..

[107] Sato T (2014). Comparison of Hansen solubility parameter of asphaltenes extracted from bitumen produced in different geographical regions. Energy Fuels.

[108] Ichihashi K (2022). Effect of the enantiomeric structure of hydrophobic polymers on the encapsulation properties of a second near infrared (NIR-II) fluorescent dye for in vivo deep imaging. RSC Adv..

[109] Garg SM (2015). Polymeric micelles based on poly(ethylene oxide) and α-carbon substituted poly(ɛ-caprolactone): an in vitro study on the effect of core forming block on polymeric micellar stability, biocompatibility, and immunogenicity. Colloids Surf. B Biointerfaces.

[110] Ueya Y (2022). Effects of hydrophilic/hydrophobic blocks ratio of PEG-b-PLGA on emission intensity and stability of over-1000 nm near-infrared fluorescence dye-loaded polymeric micellar nanoparticles. Anal. Sci..

[111] Makino A (2012). Control of in vivo blood clearance time of polymeric micelle by stereochemistry of amphiphilic polydepsipeptides. J. Control. Release.

[112] Ma C (2015). Core-shell structure, biodegradation, and drug release behavior of poly(lactic acid)/poly(ethylene glycol) block copolymer micelles tuned by macromolecular stereostructure. Langmuir.

[113] Rissanen M (2008). Solubility and phase separation of poly(L, D-lactide) copolymers. J. Appl. Polym. Sci..

[114] Zaaba NF, Jaafar M (2020). A review on degradation mechanisms of polylactic acid: hydrolytic, photodegradative, microbial, and enzymatic degradation. Polym. Eng. Sci..

